# *miR-3156-5p* is downregulated in serum of MEN1 patients and regulates expression of *MORF4L2*

**DOI:** 10.1530/ERC-22-0045

**Published:** 2022-07-07

**Authors:** Kreepa G Kooblall, Victoria J Stokes, Omair A Shariq, Katherine A English, Mark Stevenson, John Broxholme, Benjamin Wright, Helen E Lockstone, David Buck, Simona Grozinsky-Glasberg, Christopher J Yates, Rajesh V Thakker, Kate E Lines

**Affiliations:** 1 OCDEM, Radcliffe Department of Medicine, University of Oxford, Churchill Hospital, Oxford, UK; 2 Wellcome Trust Centre for Human Genetics, University of Oxford, Roosevelt Drive, Oxford, UK; 3 Neuroendocrine Tumor Unit, ENETS Center of Excellence, Endocrinology & Metabolism Department, Hadassah Medical Center and Faculty of Medicine, The Hebrew University of Jerusalem, Israel; 4 Oxford NIHR Biomedical Research Centre, Oxford University Hospitals Trust, Oxford, UK

**Keywords:** multiple endocrine neoplasia type 1, mortality factor 4-like protein 2, microRNA, neuroendocrine tumour, menin

## Abstract

Multiple endocrine neoplasia type 1 (MEN1), caused by mutations in the *MEN1* gene encoding menin, is an autosomal dominant disorder characterised by the combined occurrence of parathyroid, pituitary and pancreatic neuroendocrine tumours (NETs). Development of these tumours is associated with wide variations in their severity, order and ages (from <5 to >80 years), requiring life-long screening. To improve tumour surveillance and quality of life, better circulating biomarkers, particularly for pancreatic NETs that are associated with higher mortality, are required. We, therefore, examined the expression of circulating miRNA in the serum of MEN1 patients. Initial profiling analysis followed by qRT-PCR validation studies identified *miR-3156-5p* to be significantly downregulated (−1.3 to 5.8-fold, *P* < 0.05–0.0005) in nine MEN1 patients, compared to matched unaffected relatives. *MEN1* knock-down experiments in BON-1 human pancreatic NET cells resulted in reduced *MEN1* (49%, *P* < 0.05), menin (54%, *P* < 0.05) and *miR-3156-5p* expression (20%, *P* < 0.005), compared to control-treated cells, suggesting that *miR-3156-5p* downregulation is a consequence of loss of *MEN1* expression. *In silico* analysis identified mortality factor 4-like 2 (*MOR4FL2*) as a potential target of *miR-3156-5p*, and *in vitro* functional studies in BON-1 cells transfected with either *miR-3156-5p* mimic or inhibitors showed that the *miR-3156-5p* mimic significantly reduced MORF4L2 protein expression (46%, *P* < 0.005), while *miR-3156-5p* inhibitor significantly increased MORF4L2 expression (1.5-fold, *P* < 0.05), compared to control-treated cells, thereby confirming that *miR-3156-5p* regulates MORF4L2 expression. Thus, the inverse relationship between *miR-3156-5p* and *MORF4L2* expression represents a potential serum biomarker that could facilitate the detection of NET occurrence in MEN1 patients.

## Introduction

Multiple endocrine neoplasia type 1 (MEN1) is an autosomal dominant disorder characterised by the combined occurrence of parathyroid tumours and neuroendocrine tumours (NETs) of the pancreas and pituitary. Over 90% of patients with MEN1 have pathogenic mutations in the *MEN1* gene, which leads to loss of its encoded 610 amino acid tumour suppressor protein, menin (Brandi *et al.* 2021). Pathogenic germline and somatic *MEN1* mutations reported in both familial and sporadic cases of MEN1 are scattered throughout the nine coding exons of the *MEN1* gene and show no genotype–phenotype correlation (Lemos & Thakker 2008, Concolino *et al.* 2016, Frost *et al.* 2018, Kooblall *et al.* 2021). In addition, patients with MEN1 carry a heterozygous mutation in *MEN1*, and tumours arise when a second hit occurs, causing complete loss of functional menin protein. This results in tumours arising at different time points in different organs, even in identical twins and in individuals within the same family carrying identical mutations (Brandi *et al.* 2021, Kooblall *et al.* 2021).

Due to this variability of tumour development, *MEN1* mutation carriers are advised to undergo DNA testing, genetic counselling and regular screening for tumours, from as young as 5 years of age. Current clinical guidelines recommend annual surveillance for tumours by biochemical analyses (e.g. calcium, fasting glucose and hormones such as insulin, gastrin and prolactin) and radiological examination including MRI (or CT) scans of the pancreas and pituitary (Thakker *et al.* 2012). However, the current single secreted biomarkers for pancreatic NETs, such as gastrin, insulin and chromogranin A (CgA), have limited usefulness for diagnostic or prognostic purposes, owing to the complexity and diversity of multiple tumour development and varying responses to different therapies (Oberg *et al.* 2015). For example, serum CgA, which is constitutively secreted from neuroendocrine cells, is prone to diagnostic inaccuracies due to the current assays having a wide range in sensitivity (60–90%) and low specificity (50%) and lack of correlation with imaging techniques. Moreover, circulating CgA levels do not always correlate with tumour mass, especially when smaller tumours may be hypersecretory and larger tumours may have low secretion (Modlin *et al.* 2010, Lawrence *et al.* 2011, Yao *et al.* 2011). The importance of screening has also recently been highlighted by a study of children and adolescent MEN1 patients, which indicated that 70% of patients developed a tumour before 18 years of age, including metastatic pancreatic NETs (Shariq *et al.* 2021). Therefore, better less-invasive serum/plasma-based biomarkers are required, particularly for pancreatic NETs.

One of the possible causes of the variability of MEN1 tumour development could be the influence of epigenetic changes which can act as cofactors in driving individual MEN1 phenotypes. Thus, alteration of one or more tissue-specific epigenetic mechanisms, such as DNA methylation, histone modifications and noncoding RNAs, could affect gene expression and trigger tumour development and disease occurrence. This makes epigenetic factors suitable molecular markers for diagnostic and prognostic purposes as well as possible therapeutic targets in human diseases (Hackl *et al.* 2016, Frost *et al.* 2018). miRNA represents one type of epigenetic factor that is commonly misregulated in tumours (Frost *et al.* 2018, Donati *et al.* 2020). miRNAs are small ncRNAs that bind to target mRNAs to regulate gene expression, which can be released from tumour cells into circulation. Previous studies have shown that specific miRNA profiles can help distinguish normal, benign and malignant tissues, and miRNAs are therefore promising diagnostic and prognostic circulating biomarkers (Ardekani & Naeini 2010). Specifically, miRNAs have been reported as misregulated in MEN1-associated tumours of the parathyroids (Verdelli & Corbetta 2017), pituitary (Di Ieva *et al.* 2014) and gastroenteropancreatic (GEP) tract (Malczewska *et al.* 2018).

Thus, the identification of specific circulating miRNAs in MEN1 patients could lead to potential tumour biomarkers and possible molecular targets for therapies. We, therefore, examined the expression of miRNAs in the serum of MEN1 patients.

## Methods

### Patient information and serum collection

Serum samples, stored at −80°C, and clinical information from nine MEN1 patients (four males and five females, age range: 27–60 years) with a proven *MEN1* mutation, and their sex-matched unaffected relatives who were proven not to have *MEN1* mutations, or MEN1-associated tumours, were ascertained (Table 1). All patient and unaffected individual serum samples were processed using our standard protocol. Briefly, 5 mL of blood was collected using tubes containing no anticoagulant. Blood samples were allowed to clot and then centrifuged at 2000 ***g*** for 10 min. The resulting serum was then removed and stored at −80°C. MEN1 patient samples were divided into two cohorts: a test cohort and a validation cohort. The test cohort consisted of two males and two females (age range: 35–60 years) who had at least two MEN1-tumour manifestations at the time of blood sampling. Clinical information on all known tumours at the time of blood sampling is shown in Table 1. Thus, all test cohort patients had a parathyroid adenoma and pancreatic NET (two with gastrinomas and two with insulinomas), with one female patient also having a prolactinoma. The validation cohort consisted of five patients (two males and three females, age range: 27–56 years) who had at least one tumour manifestation at the time of blood sampling (Table 1). Thus, all validation cohort patients had a parathyroid adenoma, two patients had a pancreatic NET (one gastrinoma and one insulinoma) and one female patient had an additional prolactinoma. In both cohorts, the sex-matched unaffected relative sample was used as a control. Due to the historical nature of the samples obtained, no data on tumour size was available. Informed consent was obtained from patients and relatives using protocols approved by a UK research ethics committee (MREC/02/2/93).

**Table 1:** Patients analysed for serum miRNA changes.

	MEN1 Patient	Gender	*MEN1* mutation	~Age at time of blood sampling	Tumours present	Matched relative Non-MEN1 Control	Gender	Relationship to patient
Test	1	Male	10BP insertion (exon 2) 63-66:fs51aaX	55	Gastrinoma and parathyroid adenoma	1a	Male	Brother
	2	Male	GCT to CCT (exon 3) Ala160Pro	60	Gastrinoma and parathyroid adenoma	2a	Male	Brother
	3	Female	4BP:CAGT (exon 3) 210/211:fs11aaX	35	Insulinoma and parathyroid adenoma	3a	Female	Sister
	4	Female	1BP del T (exon 7) 327:fs53aaX	45	Insulinoma, parathyroid adenoma and prolactinoma	4a	Female	Sister
Validation	5	Female	1BP del T (exon 7) 327:fs53aaX	45	Insulinoma, parathyroid adenoma and prolactinoma	5a	Female	Sister
	6	Female	1BP del:G (exon3) 214:fs9aaX	30	Parathyroid adenoma and prolactioma	6a	Female	Sister
	7	Female	1BP del:G (exon3) 214:fs9aaX	27	Parathyroid adenoma	7a	Female	Sister
	8	Male	4BP:CAGT (exon 3) 210/211:fs11aaX	56	Parathyroid adenoma	8a	Male	Son
	9	Male	10BP ins CCAGCCCAGC (exon 2) 63-66:fs51aaX	42	Gastrinoma and parathyroid adenoma	9a	Male	Son
Surgical patient	10	Male	Glu 388 Stop	48	Parathyroidectomy, partial pancreatectomy and gastrectomy	10a	Male	Brother

### miRNA sequencing and analysis

Total RNA, including miRNAs, was extracted from 600 µL serum using the *Mir*Vana Paris Kit (Ambion). From this, the miRNA libraries were prepared using the NEBNext smallRNA kit for Illumina (E7330L) following the manufacturer’s instructions. Size selection was carried out using Blue Pippin. Individual libraries were QC’d using Tapestation (Agilent) before being pooled and sequenced on Hiseq2500 (Illumina) at the Oxford Genomics Centre (Wellcome Centre for Human Genetics, University of Oxford). For analysis, read1 of the FASTQ was trimmed using fastx_clipper (https://github.com/agordon/fastx_toolkit) and aligned using Bowtie2 (Langmead & Salzberg 2012) to GRCh37, and miRNA counts were obtained using htseq-count (Anders *et al.* 2015) against the annotation from miRBase v20. The raw gene count matrix was imported into the R/BioConductor environment (https://www.r-project.org/; Huber *et al.* 2015) for further processing and analysis with the edgeR package (Robinson *et al.* 2010, McCarthy *et al.* 2012). Genes with very low expression (i.e. those with ≤10 reads, after normalising for library size, in the four-paired samples of the test set) were excluded. Multiple testing correction was performed by using edgeR’s default Benjamini–Hochburg method for controlling the false discovery rate.

### Cell culture, transfections and functional assays

BON-1 cells, isolated from a lymph node metastasis from a pancreatic NET patient (Avniel-Polak *et al.* 2016), were cultured in Dulbecco’s Modified Eagle’s Medium (DMEM)-F12 (Gibco), supplemented with 10% fetal calf serum (FCS) (Sigma-Aldrich), and HEPG2, hepatocellular carcinoma cells, were cultured in DMEM (Gibco) with 10% FCS. Both cell lines were maintained at 37°C, with 5% (vol/vol) CO_2_. For menin knock-down experiments, 2 × 10^5^ cells were seeded into each well of six-well plates and transfected with 25 nM of either control, non-targeting (NT) siRNA or ON-TARGETplus SMARTpool of siRNAs against human *MEN1*, using Dharmafect 1 transfection reagent (all Thermo Scientific) prepared in serum-free DMEM, as described (Lines *et al.* 2018). After the addition of siRNAs, cells were incubated for 48 h, and RNA (mRNA and miRNA) or protein was harvested for further analysis. For miRNA mimic and inhibitor protein and RNA experiments, 2 × 10^5^ cells were seeded into each well of six-well plates and transfected with either 5 nM *hsa-miR-3156-5p* mimic (Qiagen) or 50 nM *hsa-miR-3156-5p* inhibitor (Qiagen), using Dharmafect 1 transfection reagent (Thermo Scientific). For controls, 5 nM of NT siRNA (Thermo Scientific) (mimic) and 50 nM control inhibitor (Qiagen) were used. After 48 h of transfection, cells were harvested for miRNA and protein analysis. For viability assays, cells were seeded at a density of 5 × 10^4^ cells/mL into black-walled 96-well plates and transfected with miR-3156-5p mimic and inhibitor or controls for 48 h. After 5 days, 20 µL Cell Titer Blue (Promega) was added to each well at 5% (vol/vol) and incubated for 1 h at 37°C before fluorescent output was read on a Pherastar Microplate reader (BMG Labtech). For apoptosis assays, cells were seeded at a density of 5 × 10^4^ cells/mL into white opaque 96-well plates and transfected with *miR-3156-5p* mimic and inhibitor or controls for 48 h. After 5 days, 75 µL of Caspase 3/7 Glo reagent (Promega) was added per well, incubated for 1 h at room temperature and the luminescent outputs were read on a Pherastar Microplate reader (BMG Labtech). For wound-healing migration assays, cells were seeded at a density of 5 × 10^4^ cells/mL into 24-well plates and transfected with *miR-3156-5p* mimic and inhibitor or controls for 48 h. Wounds were made in the cell monolayer using a pipette tip and pictures were taken using a light microscopes, ×10 magnification, and after 3 days, images were taken again for comparison. Migration was assessed by measuring the size of the wound using Image J software and subtracting the size of the day 3 wound from the day 0 wound.

### Quantitative reverse transcription PCR (qRT-PCR)

Total RNA was extracted from patient’s serum or BON-1 cells using the *miR*Vana Paris kit (Ambion), and 1 µg was used to generate cDNA using the MiScript RT kit (Qiagen). Quantitect primers (Qiagen) were used for mRNA qRT-PCR reactions, and miScript primers (Qiagen) for miRNA reactions, which utilised the Quantitect SYBR green kit (Qiagen), on a RotorGene 5, as described (Lines *et al.* 2017). Each test sample was normalized to the geometric mean of reference genes Glyceraldehyde 3-phosphate dehydrogenase (*GAPDH*) and α-tubulin (*TUBA1A*) (for mRNA), or RNA, U6 Small Nuclear 6 (*RNU6B*) and snoRNA, C/D Box 95 (*SNORD95*) (for miRNA’s). Serum samples were spiked with 20 fmol *cel-miR-39-3p* (Qiagen) prior to miRNA extraction as a reference control. Analysis of serum samples was performed on miRNAs extracted on a minimum of three separate occasions from different frozen aliquots, and cell line studies were performed in *n*  = 4 biological replicates. The relative expression of target cDNA in all qRT-PCR studies was determined using the Pfaffl method (Pfaffl 2001).

### Western blot analysis

NET cells were lysed in NP40 lysis buffer and prepared in 4× Laemmli loading dye (BioRad) boiled at 95°C for 5 min, resolved using 6% or 10% SDS-PAGE gel electrophoresis, and transferred to PVDF membrane, as described (Lines *et al.* 2017). Membranes were probed with the primary antibodies rabbit anti-menin, rabbit-anti mortality factor 4-like 2 (MORF4L2), rabbit-anti GAPDH or rabbit-anti calnexin (all Abcam) and anti-rabbit horseradish peroxidase (HRP)-conjugated secondary antibody (Santa Cruz Biotechnology), as described (Lines *et al.* 2017). Blots were visualised using Pierce ECL Western blotting substrate (Thermo Fisher Scientific), as described (Lines *et al.* 2017). GAPDH or calnexin protein expression was determined as a loading control. Densitometry analysis was performed by calculating the number of pixels per band using ImageJ software. Data were represented as the number of pixels of the protein band, relative to the number of pixels of the corresponding GAPDH or calnexin band.

### *In silico* analysis

The miRNA target prediction database (miRDB) (http://mirdb.org) (Chen & Wang 2019, Liu & Wang 2019) was examined for potential targets of the miRNA *miR-3156-5p*. Targets were searched using the term ‘miR-3156-5p’ and limited to human targets only. The database was last accessed on 5 August 2021.

### Statistical analysis

Data were analysed using Student’s *t*-test where there were only 2 groups or using 1-way ANOVA using a Bonferroni correction for multiple comparisons where there were >2 groups.

## Results

### miRNAs are dysregulated in the serum of MEN1 patients

Profiling analysis of the test cohort of four MEN1 patients (two females and two males) identified five miRNAs that were upregulated, and six miRNAs that were downregulated in the serum of all MEN1 patients, compared to their matched control relative (Table 2). These miRNAs were all dysregulated by greater than 2-fold and *P* < 0.05. The two most highly upregulated miRNAs were *miR-125a-3p* (4.38-fold, *P* = 0.03) and *miR-582-3p* (4.06-fold, *P* = 0.04), and the two most highly downregulated miRNAs were *miR-3156-5p* (−11.62-fold, *P* = 0.02) and *miR-3168* (−3.66-fold, *P* = 0.01) (Table 2).

**Table 2:** Dysregulated miRNAs in four test MEN1 patients compared to unaffected relatives. Data are sorted by fold change and are represented as an average of the fold change occurring in the 4 MEN1 patients.

		Fold change	LogCPM	*P*-value
Upregulated	*hsa-miR-125a-3p*	4.38	5.78	0.03
	*hsa-miR-582-3p*	4.06	6.52	0.04
	*hsa-miR-654-5p*	3.45	5.93	0.05
	*hsa-miR-335-5p*	3.37	8.80	0.04
	*hsa-miR-215*	2.81	9.07	0.04
Downregulated	*hsa-miR-107*	−2.79	10.15	0.02
	*hsa-miR-501-3p*	−2.65	8.85	0.04
	*hsa-miR-92a-3p*	−2.72	16.13	0.01
	*hsa-miR-9-5p*	−2.87	7.85	0.04
	*hsa-miR-3168*	−3.66	8.40	0.01
	*hsa-miR-3156-5p*	−11.62	6.89	0.02

LogCPM, log2 of counts per million reads.

### *miR-3156-5p* is downregulated in the serum of MEN1 patients

**Figure 1:**
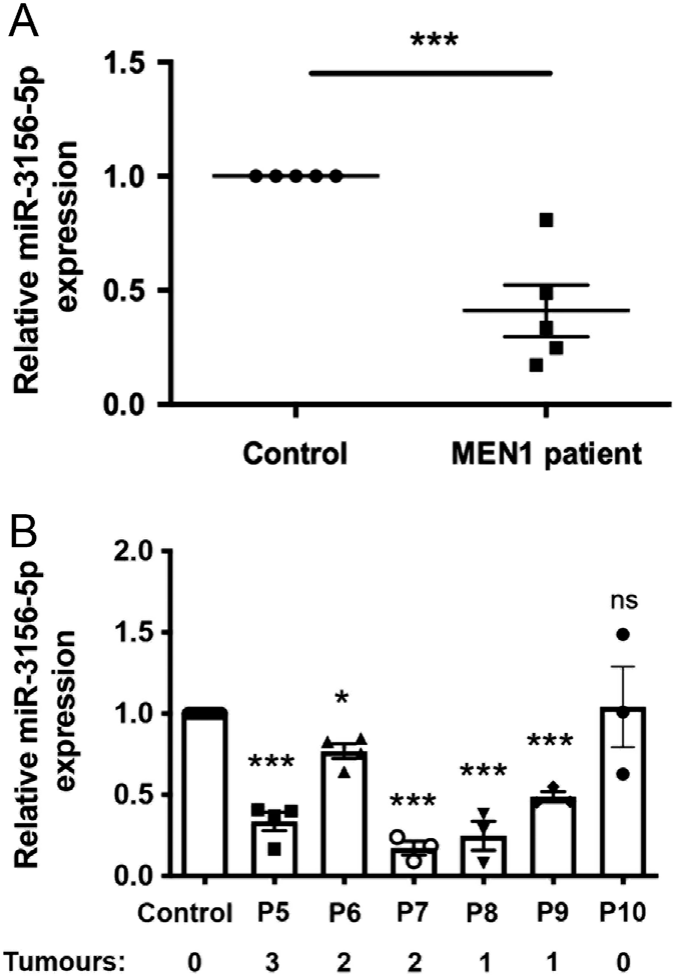
*miR-3156-5p* downregulation in serum of MEN1 patients. *miR-3156-5p* expression was reduced in the serum of five MEN1 patients, when compared to sex-matched control (unaffected) relatives (A). *n*  = 3-4 technical replicates were undertaken for each patient and controls with *n*  = 5 biological replicates. A *t*-test was used to determine significance; ***P < 0.005. Data on all five patients (P5-9, Table 1) was then assessed individually, with samples arranged by the number of MEN1-associated tumour manifestations, to determine if all showed statistically significant reductions in *miR-3156-5p* compared to controls. A patient (P10) who had undergone extensive treatment and was described as tumour-free has also been included for comparison (B). *n*  = 3-4 technical replicates were undertaken for each patient and control. One-way ANOVA was undertaken to determine statistical significance; *P < 0.05, ***P < 0.0005.

The observed up- and downregulation of the miRNAs from our sequencing analysis (Table 2) were confirmed using qRT-PCR analysis in samples from a validation cohort of an additional five MEN1 patients (three females and two males) and sex-matched unaffected relatives (Table 1). This showed that *miR-3156-5p* was significantly downregulated by 2.4-fold (*P* < 0.005) in the serum of MEN1 patients, when compared to the unaffected control relatives (Fig. 1A). Moreover, all five MEN1 patients also demonstrated a significant decrease in *miR-3156-5p*, when compared to their unaffected control relative, with a range of 1.3-fold (*P* < 0.05) to 5.8-fold (*P* < 0.0005) (Fig. 1B). Analysis of *miR-3156-5p* expression in a MEN1 patient who had undergone significant treatment to remove their tumours, including parathyroidectomy, pancreatectomy and gastrectomy, indicated there to be no significant difference when compared to their unaffected control relative (P10, Fig. 1B). Significant alterations were not consistently observed in the expression of miRNAs *miR-125a-3p*, *miR-582-3p* or *miR-3168* (Supplementary Fig. 1, see section on supplementary materials given at the end of this article).

### *miR-3156-5p* expression is downregulated after *MEN1* knock-down

**Figure 2:**
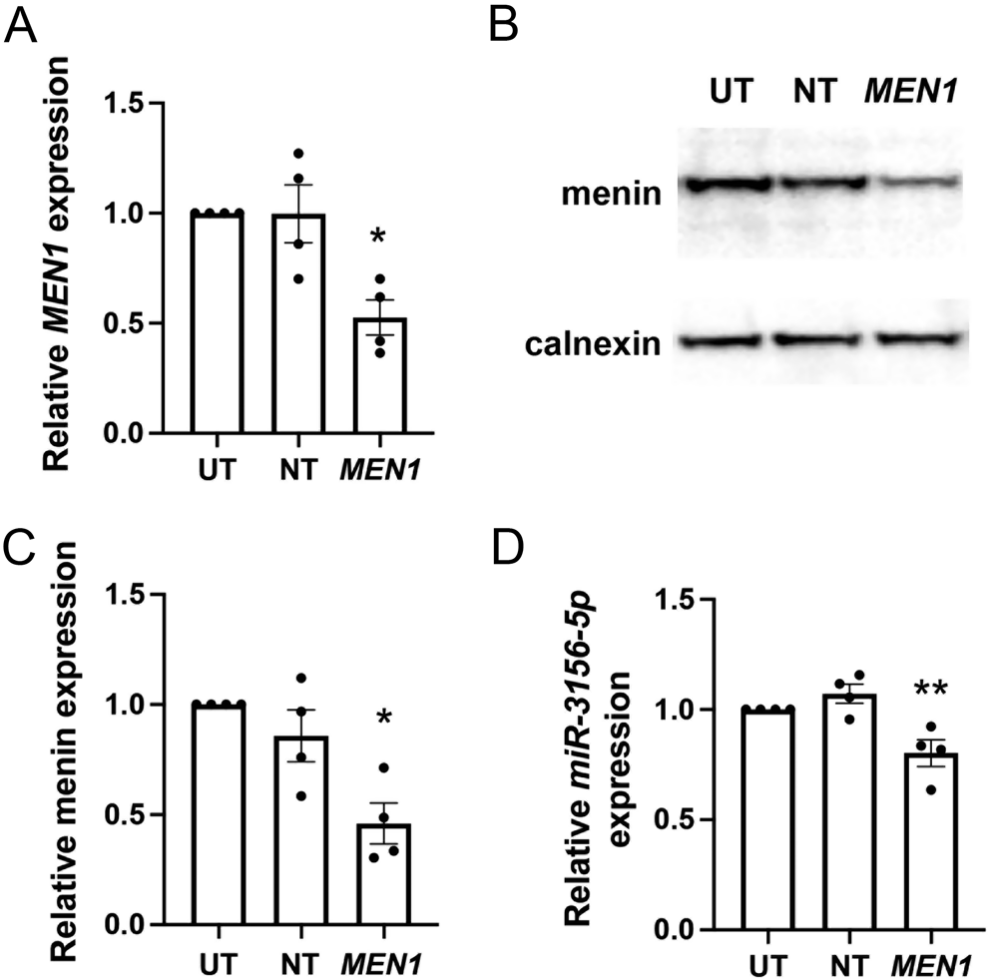
Expression of *miR-3156-5p* after menin knock-down. BON-1 cells were treated with siRNA for *MEN1* or control non-targeting (NT) siRNA. Untransfected (UT) cells were used as controls. Quantitative RT PCR (qRT-PCR) was used to evaluate *MEN1* expression (A). Data are represented relative to UT cells. Studies were undertaken in *n*  = 4 biological replicates. Statistical significance comparing all treatments to each other was assessed by one-way ANOVA; *P < 0.05. Menin protein, encoded by *MEN1*, was assessed by Western blot analyses, with calnexin (housekeeper) used as a control (B). A representative Western blot is shown. Protein expression from the Western blot was quantified using densitometry analysis from *n*  = 4 biological replicates (C). Data are represented relative to UT cells. Statistical significance comparing all treatments to each other was assessed by one-way ANOVA; *P < 0.05. *miR-3156-5p* expression after *MEN1* knock-down was evaluated using qRT-PCR (D). Data are represented relative to UT cells. Studies were undertaken in *n*  = 4 biological replicates, and statistical significance comparing all treatments to each other was assessed by one-way ANOVA; **P < 0.005.

To determine whether the downregulation of *miR-3156-5p* was a consequence of loss of *MEN1* expression, we undertook *MEN1* knock-down experiments in the menin-expressing pancreatic NET cell line, BON-1. Knock-down after 48 h was confirmed by qRT-PCR, which showed a decrease in *MEN1* mRNA by 49% (*P* < 0.05), compared to control NT siRNA controls (Fig. 2A), and Western blot analysis, which showed a 54% reduction (*P* < 0.05) in the expression of menin (Fig. 2B and C). This menin knock-down was associated with a 20% (*P* < 0.005) decrease in *miR-3156-5p* expression when compared to control-treated cells (Fig. 2D).

### *MORF4L2* is a target of *miR-3156-5p*

The biological consequences of the downregulation of *miR-3156-5p* expression were investigated using an *in silico* approach to identify potential target genes, and an examination of the miRDB (http://mirdb.org) database revealed a total of 353 predicted targets. The top 25 ranked targets (Table 3) comprised genes involved in a wide range of cellular activities including regulation of gene transcription (e.g. pleiomorphic adenoma gene 1 zinc finger (*PLAG1*)*,* promyelocytic leukaemia nuclear body scaffold (*PML*)*,* RNA polymerase III subunit G (*POLR3G*), zinc finger protein 273 (*ZNF273*)*,* 5’-3’ exoribonuclease 1 (*XRN1*)*,* zinc finger protein 711 (*ZNF711*)and CCR4-NOT transcription complex subunit 7 (*CNOT7*)); ion transport (e.g. ChaC glutathione-specific gamma-glutamylcyclotransferase 2 (*CHAC2*), and potassium inwardly rectifying channel subfamily J member 16 (*KCNJ16*)); ras signalling (e.g. Ras-related protein rap-1a, member of ras oncogene family (*RAP1A*), and Ras-related protein rab-27a, member of ras oncogene family (*RAB27A*)) and cell-cycle regulation (e.g. cell division cycle 123 (*CDC123*)and Syndecan 2 (*SDC2*)). In addition, mortality factor 4-like 2 (*MOR4FL2*) was identified as a potential target. This was of particular interest as this transcript has been described as part of the neuroendocrine neoplasms test (NETest), which consists of 51 transcripts that are upregulated in NETs, and is used as a blood biomarker test for the management and diagnosis of multiple NET subtypes including GEP and bronchopulmonary NETs (Modlin *et al.* 2014, 2016, 2018).

**Table 3:** Predicted gene targets of *miR-3156-5p* according to miRDB (http://mirdb.org). In total, there were 353 predicted targets, the top 25 of which are shown, ranked by their assigned target score.

Target rank	Target score	Gene symbol	Gene description
1	97	*DIPK2A*	Divergent protein kinase domain 2A
2	96	*CDC123*	Cell division cycle 123
3	96	*SESTD1*	SEC14 and spectrin domain containing 1
4	95	*PLAG1*	PLAG1 zinc finger
5	95	*C3orf38*	Chromosome 3 open reading frame 38
6	94	*KLHL11*	Kelch like family member 11
7	93	*CHAC2*	ChaC cation transport regulator homolog 2
8	93	*KCNJ16*	Potassium voltage-gated channel subfamily J member 16
9	93	*PML*	Promyelocytic leukaemia
10	93	*RAP1A*	RAP1A, member of RAS oncogene family
11	93	*POLR3G*	RNA polymerase III subunit G
12	92	*RAB27A*	RAB27A, member RAS oncogene family
13	91	*ROCK2*	Rho-associated coiled-coil containing protein kinase 2
14	91	*BCAT1*	Branched chain amino acid transaminase 1
15	91	*ZNF273*	Zinc finger protein 273
16	90	*SDC2*	Syndecan 2
17	90	*XRN1*	5’-3’ exoribonuclease 1
18	90	*TACC1*	Transforming acidic coiled-coil-containing protein 1
19	90	*NIPAL2*	NIPA-like domain containing 2
20	90	*LYZ*	Lysozyme
21	89	*ZNF711*	Zinc finger protein 711
22	89	*CNOT7*	CCR4-NOT transcription complex subunit 7
23	88	*SDF2*	Stromal cell-derived factor 2
24	88	*SKAP2*	Src kinase-associated phosphoprotein 2
25	88	*MORF4L2*	Mortality factor 4-like 2

### *MORF4L2* expression is regulated by *miR-3156-5p*

**Figure 3:**
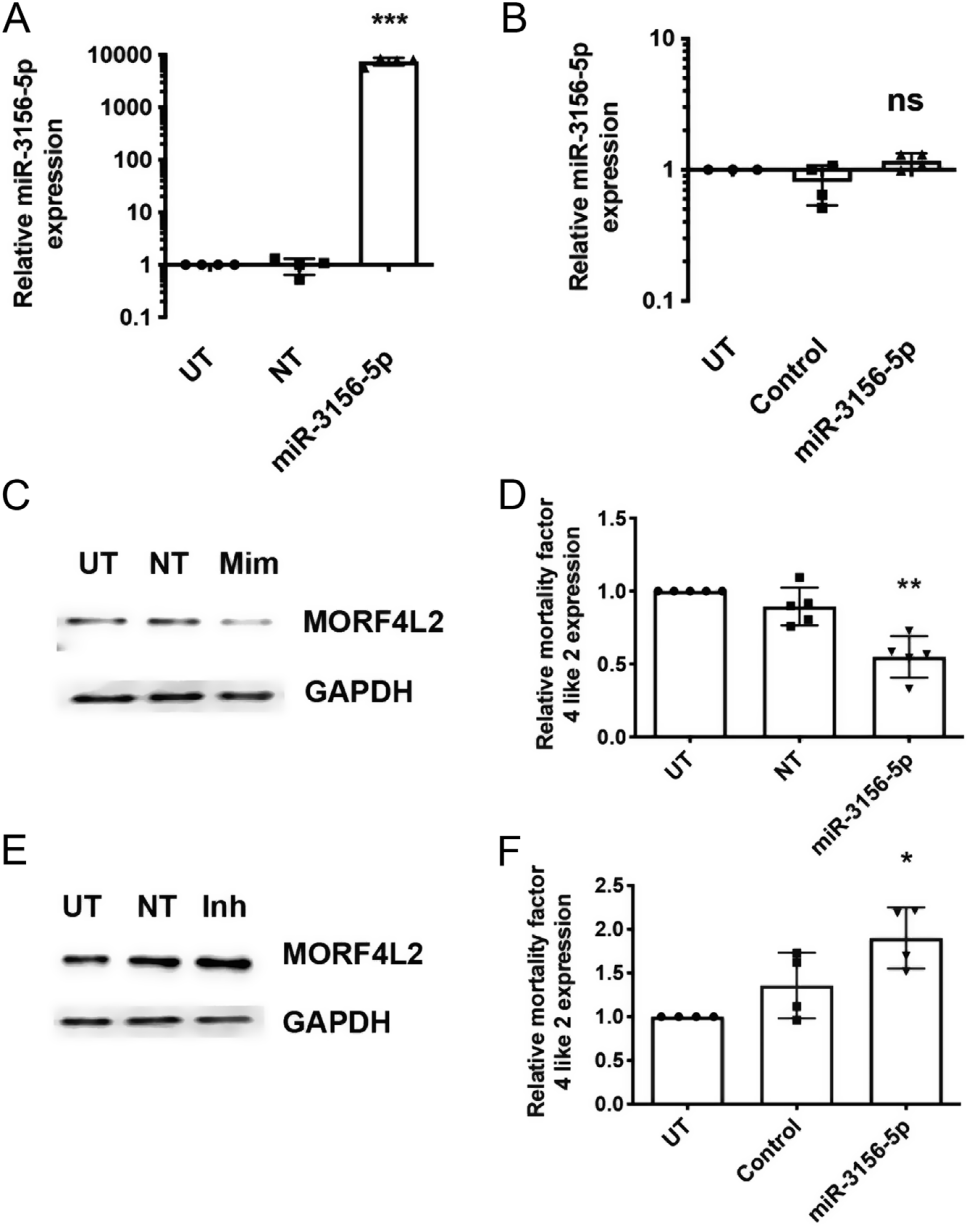
Regulation of MORF4L2 expression by *miR-3156-5p.* BON-1 cells were transfected with either *miR-3156-5p* mimic or inhibitors for 48 h, and MORF4L2 expression was assessed. Confirmation of *miR-3156-5p* mimic transfection was undertaken using qRT-PCR (A). Untransfected (UT) cells and non-targeting (NT) scrambled RNA-treated cells were used as controls. Experiments were performed in *n*  = 4 biological replicates. Data are represented relative to UT cells, with statistical significance comparing all transfections to each other assessed by one-way ANOVA; ***P < 0.0001. To determine if inhibitor transfection altered *miR-3156-5p* expression, qRT-PCR analysis was undertaken (B). UT and control miRNA inhibitor transfections were used as controls. Experiments were performed in *n*  = 4 biological replicates. Data are represented relative to UT cells, with statistical significance comparing all transfections to each other assessed by one-way ANOVA; ns, not significant. MORF4L2 expression after mimic treatment was assessed by Western blot analyses (C). A representative image from *n*  = 4 biological replicates is shown. GAPDH was used as a housekeeper. Western blot analyses were quantified using densitometry analysis (D). Data are represented relative to UT cells. Studies were undertaken in *n*  = 4 biological replicates, and statistical significance comparing all treatments to each other was assessed by one-way ANOVA; **P < 0.005. MORF4L2 expression after inhibitor treatment was also assessed by Western blot analyses (E). A representative image from *n*  = 4 biological replicates is shown. GAPDH was used as a housekeeper. The Western blots were quantified using densitometry analysis (F). Data are represented relative to UT cells. Studies were undertaken in *n*  = 4 biological replicates, and statistical significance comparing all treatments to each other was assessed by one-way ANOVA; *P < 0.05.

**Figure 4:**
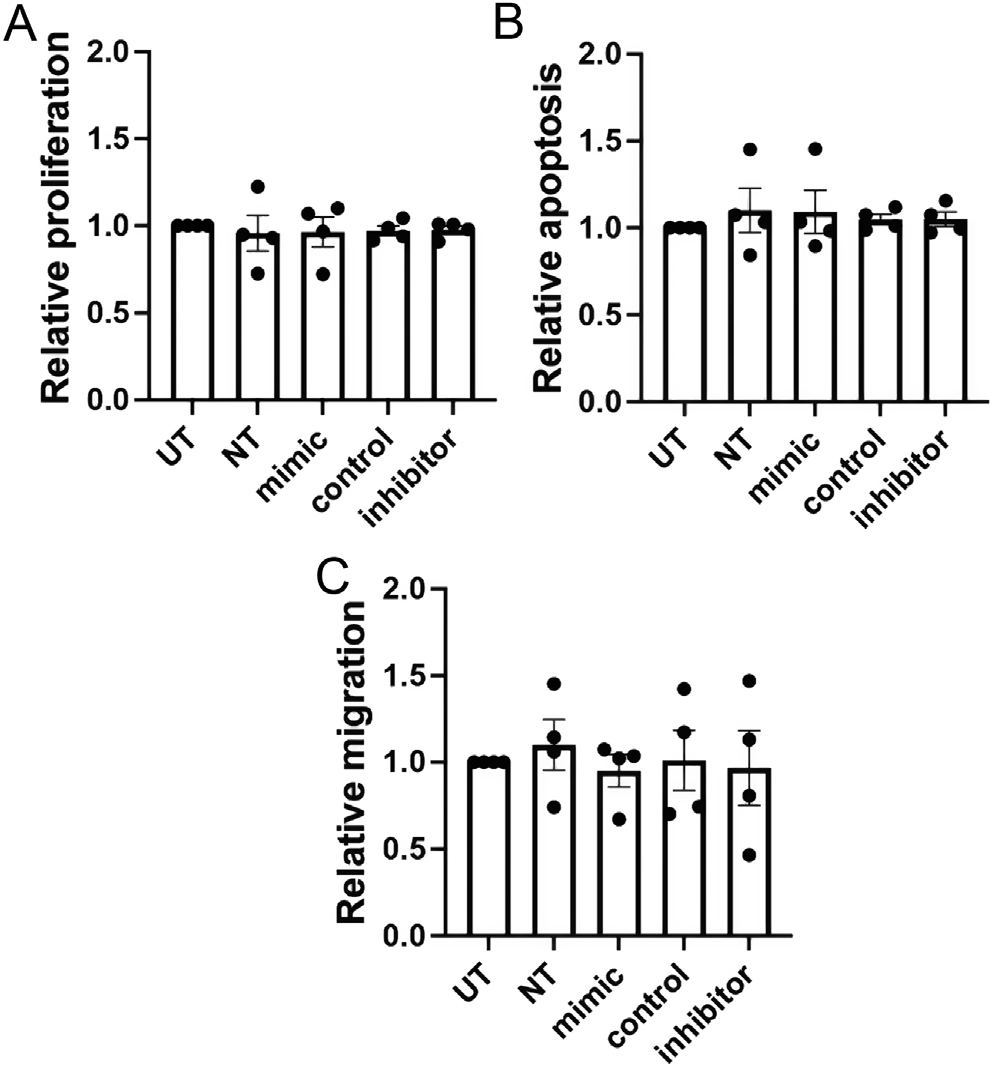
Functional analysis after dysregulation of *miR-3156-5p* in BON-1 cells. BON-1 cells were transfected with either *miR-3156-5p* mimic or inhibitors for 48 hours, untransfected (UT) cells and non-targeting (NT) scrambled RNA-treated cells or control inhibitor-treated cells were used as controls. All experiments were performed in *n*  = 4 biological replicates. Cell viability was assessed 5 days after transfection using Cell Titer blue assay. Data are shown relative to UT cells. Statistical analysis using one-way ANOVA indicated no significant difference (A). Apoptosis was assessed using Caspase 3/7 Glo assay 5 days post-transfection. Data are shown relative to UT cells. Statistical analysis using one-way ANOVA indicated no significant difference (B). Cell migration was assessed 5 days post-transfection using wound-healing assays. Data are shown relative to UT cells. Statistical analysis using one-way ANOVA indicated no significant difference (C).

**Figure 5:**
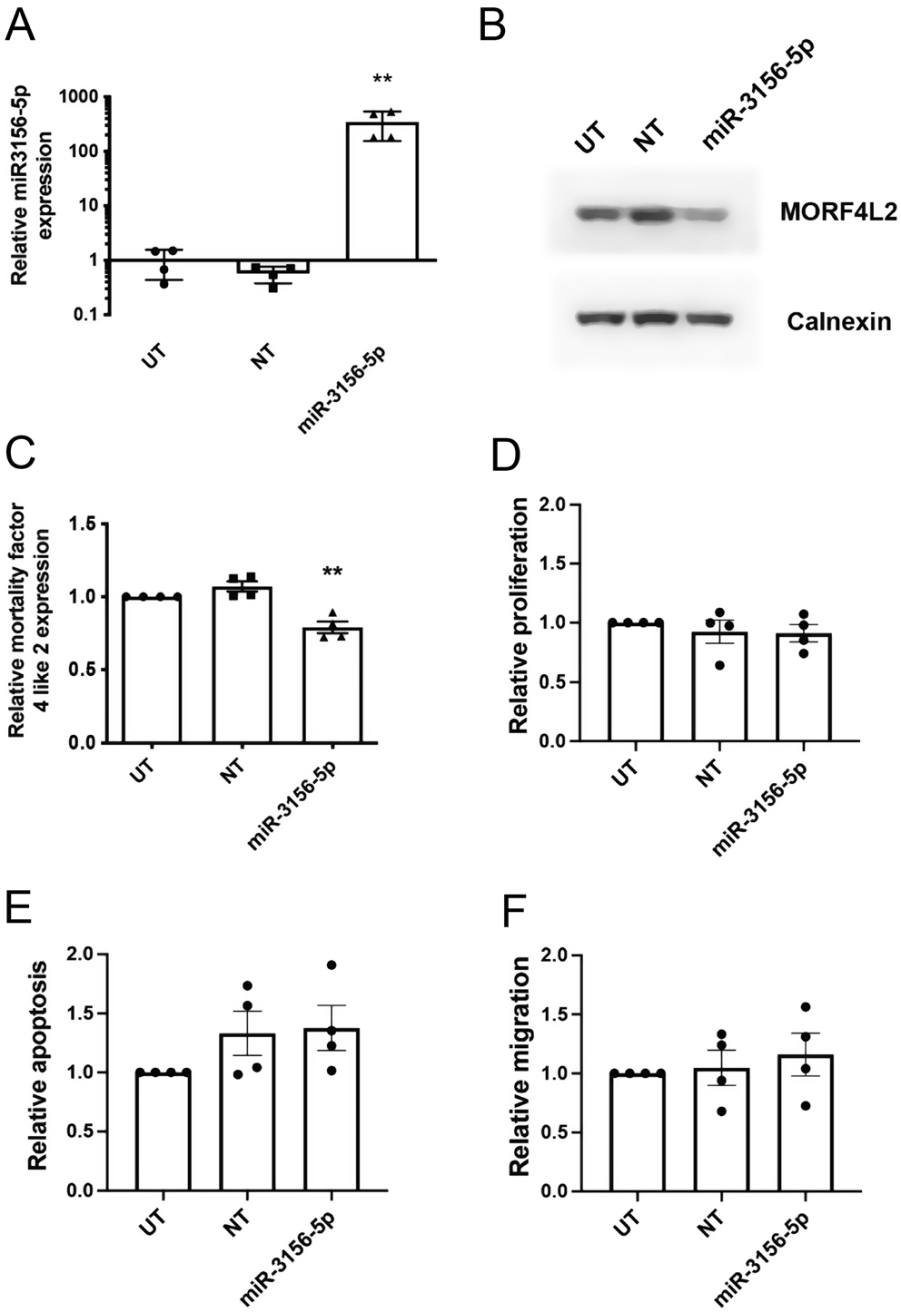
*m* *iR* *-3156-5p* regulates MORF4L2 in HEPG2 cells. HEPG2 cells were transfected with *miR-3156-5p* mimic for 48 h. Untransfected (UT) cells and non-targeting (NT) scrambled RNA-treated cells were used as controls. Experiments were performed in *n*  = 4 biological replicates. Confirmation of *miR-3156-5p* mimic transfection was undertaken using qRT-PCR. Data are represented relative to UT cells. Statistical analysis was performed using one-way ANOVA, **P < 0.005 (A). MORF4L2 expression after mimic treatment was assessed by Western blot analyses. A representative image from *n*  = 4 biological replicates is shown. Calnexin was used as a housekeeper (B). Western blot analyses were quantified using densitometry. Statistical analysis was performed using one-way ANOVA, **P < 0.005 (D). After 5 days of transfection, the effect of *miR-3156-5p* overexpression was assessed in cell viability (E), apoptosis (F) and migration assays (G). No significant difference was observed in any of the three assays.

To investigate the role of *miR-3156-5p* in regulating the expression of MORFL2, we transfected BON-1 cells with miRNA mimics and inhibitors and assessed for alterations in MORF4L2 transcripts and protein. Successful transfection of a *miR-3156-5p* mimic was confirmed using qRT-PCR, which demonstrated a 7566-fold increase (*P* < 0.0001) in *miR-3156-5p* expression (Fig. 3A). Expression of *miR-3156-5p* was not altered after inhibitor treatment (Fig. 3B), but this was to be expected as the inhibitor blocks the activity of the miRNA by complementary binding, thereby removing the ability of *miR-3156-5p* to bind to its target mRNAs, rather than by reducing its expression. Transfection with the *miR-3156-5p* mimic significantly reduced MORF4L2 protein expression, assessed by Western blot and densitometry quantification, by 46% (*P* < 0.005), when compared to control-treated cells (Fig. 3C and D). Treatment with *miR-3156-5p* inhibitor significantly increased MORF4L2 expression 1.5-fold when compared to control (*P* < 0.05) (Fig. 3E and F). To determine whether changes in *miR-3156-5p* affected BON-1 cellular function, we also undertook cell viability (Fig. 4A), apoptosis (Fig. 4B) and migration assays (Fig. 4C) after *miR-3156-5p* mimic and inhibitor treatment. This showed no significant differences. To confirm these mechanistic insights into the regulation of MORF4L2, we also transfected HEPG2 cells with *miR-3156-5p* mimic. Successful transfection resulting in a 346-fold increase (*P* < 0.005) in *miR-3156-5p* expression was confirmed by qRT-PCR (Fig. 5A). This increased *miR-3156-5p* expression caused a 21% (*P* < 0.005) decrease in MORF4L2 expression, as shown by Western blot (Fig. 5B and C). Similar to BON-1 cells, this change in *miR-3156-5p* and MORF4L2 expression did not result in changes in cell viability (Fig. 5D), apoptosis (Fig. 5E) or cell migration (Fig. 5F).

## Discussion

Our studies have revealed that *miR-3156-5p* is significantly downregulated in the sera of MEN1 patients (Fig. 1 and Table 2) and that this downregulation of *miR-3156-5p* may be a direct result of reduced *MEN1* expression (Fig. 2). *miR-3156-5p* is a human-specific mature miRNA that is processed from the transcribed stem-loop *miR-3156-2*, the sequence for which is located within an intron of the ankyrin repeat domain 30B (*ANKRD30B*) on chromosome 18. The potential utility of *miR-3156-5p* as a serum biomarker has been investigated in patients with breast, colorectal, lung, thyroid and melanoma tumours (Ferracin *et al.* 2015). For example, *miR-3156-5p* was reported to be significantly upregulated in the tumour tissue of patients with metastatic colorectal cancer, who responded to treatment with bevacizumab/5-flourouracil, leucovorin, oxaliplatin (FOLFOX), when compared to tumour tissue from non-responding patients (Kiss *et al.* 2017). Moreover, *miR-3156-5p* in combination with three other predictive miRNAs correctly identified responders to the bevacizumab/FOLFOX therapy with 82% sensitivity and 64% specificity (Kiss *et al.* 2017). In breast cancer, *miR-3156-5p* has been reported to target the proapoptotic gene, Caspase 2 (*CASP2*) and the long ncRNA tumour protein translationally controlled 1 (TPT1) antisense RNA 1(TPT1-AS1), thereby facilitating TPT1-AS1 inhibition of cell proliferation and sensitisation of breast cancer cells to chemotherapy (Huang *et al.* 2021). Based on these studies, the application of using *miR-3156-5p*, in combination with other biomarkers, represents a novel approach to evaluating cancer progression, prognosis and sensitivity to treatment.

In our study, we demonstrated *miR-3156-5p* to be consistently significantly downregulated in the sera of nine individual MEN1-patients, compared to sex-matched unaffected control relatives (Fig. 1). All patients had a parathyroid adenoma; however, each patient exhibited different tumour manifestations, ranging from one MEN1-associated tumour to three MEN1-associated tumours (Table 1). However, our study consists of small sample size, and due to the historical nature of the samples collected, only limited samples and clinical data were available. Therefore, our data do not take into account tumour size, ongoing treatments or *miR-3156-5p* and MORF4L2 expression levels with the tumours. A larger, prospective study would therefore be required to determine whether *miR-3156-5p* either alone or in combination (i.e. with existing biomarkers, e.g., chromogranin A, or theNETest, or hormone levels, e.g., insulin) could be a reliable MEN1-associated NET biomarker and whether this would correlate with disease burden. This could include recruiting patients with different types of NETs, for example, pancreatic vs thoracic NETs, as well as subgroups of MEN1 patients, for example, those without NETs, those with small <2 cm NETs and those with metastatic lesions, as well as those with syndromes including Zollinger–Ellison syndrome.

Our *in silico* analysis (Table 3) identified *MORF4L2* as a potential predicted gene target of *miR-3156-5p*, and our *in vitro* functional studies in BON-1 and HEPG2 cells confirmed that *miR-3156-5p* regulates MORF4L2 expression (Figs 3 and 5). MORF4L2 is a component of the NuA4 histone acetyltransferase complex, which catalyses the acetylation of histone H2A and H4 tails. This nucleosomal modification alters the interaction between DNA, histones and other proteins that facilitate the transcriptional activation of selected genes involved in the activation of oncogene and proto-oncogene-mediated growth induction, tumour suppressor-mediated growth arrest, replicative senescence, suppressed apoptosis and DNA repair (Kuete *et al.* 2012). In our studies, *miR-3156-5p* expression changes did not alter cell viability, apoptosis or cell migration (Figs 4 and 5). This is likely because key oncogenic changes have already occurred in these cell lines to drive these mechanisms. MORF4L2 is also a component of the NETest, which is reported to be of use in the management and diagnosis of multiple NET subtypes. The NETest, which is a blood biomarker test that comprises 51 transcripts that are upregulated in NETs (Modlin *et al.* 2014, 2016, 2018), is reported to be the highest predictive assessment method for NET disease status and progression (69%), when compared to other single secreted NET biomarkers assays, such as CgA (13%) (Modlin *et al.* 2014). For example, the NETest is reported to have a sensitivity of >95% and specificity of >90%, making it more accurate than CgA at monitoring NET disease occurrence, progression and response to therapies (Modlin *et al.* 2018). Although developed for sporadic NETS, this NETest can detect multiple NET subtypes and therefore may have utility in MEN1 patients. However, MEN1 patients commonly have concurrent tumours, and it seems that modifications to the NETest are likely to be required to improve its diagnostic use in such patients. Moreover, currently, the measurement of miRNAs in the circulation remains complex and is not available as a standardised assay in clinical practice (Kidd *et al.* 2015, Oberg *et al.* 2015). However, the use of matched miRNA and transcript data, such as *miR-3156-5p* and *MORF4L2* respectively, may be a way of refining and improving currently available biomarker tests for monitoring NET disease and progression in MEN1 patients, as it would be expected that *miR-3156-5p* levels would decrease while MORF4L2 levels simultaneously increase with increasing tumour burden. Thus, longitudinal analysis of *miR-3156-5p* and *MORF4L2* within an individual MEN1 patient could provide important information on when tumour development has occurred and aid in determining the appropriate timing to initiate more invasive screening methods.

Our data indicating that menin can regulate *miR-3156-5p* (Fig. 2), which in turn can regulate MORF4L2 expression (Fig. 3), provides additional novel insights into the importance of miRNA regulation in NET development. A role for miRNAs in the regulation of menin expression and in the development of MEN1-associated tumours has previously been reported. For example, in a *Men1* knockout mouse model, loss of cell-cycle control and pituitary tumourigenesis were associated with *miR-15a*, *miR-16-1* and *let-7a* downregulation and cyclin D1 upregulation in pituitary adenomas compared to normal WT pituitaries (Lines *et al.* 2018). Furthermore, *in vitro* functional studies in AtT20 mouse pituitary cell lines transfected with *Men1* siRNA confirmed that loss of menin expression resulted in decreased *miR-15a* expression (Lines *et al.* 2018). In addition, overexpression of *miR-17* has been shown to promote pancreatic beta cell proliferation by downregulating menin expression in the MIN6 mouse insulinoma-derived pancreatic beta cell line (Lu *et al.* 2015). Studies of human parathyroid adenomas have also demonstrated negative feedback between *MEN1* mRNA, menin and* miR-24-1*, whereby *miR-24-1* silences menin expression post-transcriptionally to mimic the second hit of Knudson’s model of tumourigenesis (Luzi *et al.* 2016). Parathyroid adenomas from MEN1 patients with a heterozygous *MEN1* mutation are also reported to have reduced *MEN1* mRNA levels, lack menin expression and overexpress *miR-24-1*, despite the presence of one WT *MEN1* allele (Luzi *et al.* 2012). More recently, studies have reported additional miRNAs (such as *miR-28*, *miR-4258*, *miR-1301* and *miR-664*) as potential mediators of MEN1 parathyroid tumourigenesis by similarly silencing *MEN1* or other tumour suppressor genes, such as *CCND1*, *RET*, *CDKN1B*, *RB1*, *VDR*, *PRDM2*, *CDKN2C* and *CDC73* (Grolmusz *et al.* 2017, Luzi *et al.* 2017). Thus, the interaction of miRNAs and menin may be crucial in regulating and monitoring NET development and could provide novel biomarker panels for MEN1 patients. For example, a biomarker panel consisting of multiple dysregulated miRNAs with their corresponding target proteins (e.g. *miR-3156-5p* and MORF4L2) could provide blood biomarkers with high sensitivity and specificity. This approach could also be utilised to identify novel miRNA–protein target combinations that act as specific biomarkers for different tumours, including gender-specific tumours, for example, thymic carcinoids in males and bronchial carcinoids in females, which will also help inform on the underlying biology.

In summary, our results, which reveal an inverse relationship between *miR-3156-5p* and MORF4L2 expression, may help to increase the reliability of non-invasive blood biomarkers for the diagnosis, progression and treatment outcomes of NETs in MEN1 patients.

## Supplementary Material

Supplementary Figure 1. Expression of candidate miRNAs in a validation cohort of 5 MEN1 patients. Quantitative reverse transcription-PCR (qRT-PCR) was undertaken on the serum of an additional 5 MEN1 patients, with data shown relative to that of a sex matched unaffected relative. The two most highly upregulated miRNAs in the profiling experiment (miR-125a-3p and miR-582-3p) are indicated in red, and the second most highly downregulated miRNA (miR-3168) is indicated in green. A significant alteration in expression in these miRNAs was not observed. All experiments were undertaken with n=4 technical replicates.

## Declaration of interest

The authors declare that there is no conflict of interest that could be perceived as prejudicing the impartiality of the research reported.

## Funding

This work was supported by: the UK Medical Research Council http://dx.doi.org/10.13039/501100000265 (MRC) grants G9825289 and G1000467 (K E L, M S, R V T); AMEND Research Fund Award (K E L); a Wellcome Trust http://dx.doi.org/10.13039/100010269 Senior Investigator Award (R V T); Royal Australasian College of Physicians http://dx.doi.org/10.13039/501100001232 Vincent Fairfax Family Foundation Research Fellowship (C J Y); Australia Awards http://dx.doi.org/10.13039/501100001137 Endeavour Postgraduate Research Fellowship Award (C J Y); Nova http://dx.doi.org/10.13039/501100010450rtis Pharmaceuticals Australia Educational Grant (C J Y); Ipsen http://dx.doi.org/10.13039/501100014382 Pharmaceuticals Australia Educational Grant (C J Y); and The Unicorn Foundation Educational Grant (C J Y). National Institute for HealthResearch (NIHR) Senior Investigator Award (R V T); and NIHR Oxford Biomedical Research Centre http://dx.doi.org/10.13039/501100013373 Programme (R V T).
