# Supplementary material for: miR-3156-5p is downregulated in serum of MEN1 patients and regulates expression of MORF4L2
Source: Endocr Relat Cancer. 2022 Jul 7;29(10):557–68. doi: 10.1530/ERC-22-0045 (PMC9422251; doi:10.1530/ERC-22-0045)
Supplement: Supplementary Figure 1. Expression of candidate miRNAs in a validation cohort of 5 MEN1 patients. Quantitative reverse transcription-PCR (qRT-PCR) was undertaken on the serum of an additional 5 MEN1 patients, with data shown relative to that of a sex matched unaffected relative. The two most highly  [file supplementary_figure_1.pdf]

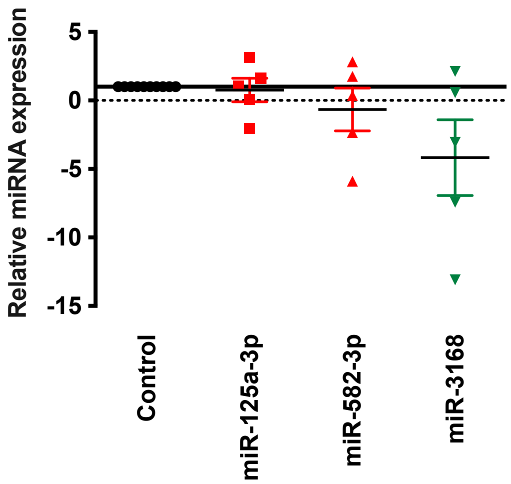

Supplementary Figure 1. Expression of candidate miRNAs in a validation cohort of 5 MEN1 patients. Quantitative reverse transcription-PCR (qRT-PCR) was undertaken on the serum of an additional 5 MEN1 patients, with data shown relative to that of a sex matched unaffected relative. The two most highly upregulated miRNAs in the profiling experiment (miR-125a-3p and miR-582-3p) are indicated in red, and the second most highly downregulated miRNA (miR-3168) is indicated in green. A significant alteration in expression in these miRNAs was not observed. All experiments were undertaken with n=4 technical replicates.
